# Prophylaxis With Oral Zinc Sulfate Against Radiation Induced Oral Mucositis in Patients With Head and Neck Cancers: A Systematic Review and Meta-Analysis of Four Randomized Controlled Trials

**DOI:** 10.3389/fonc.2019.00165

**Published:** 2019-03-22

**Authors:** Ting Shuai, Xu Tian, Bing Shi, Hui Chen, Xiao-Ling Liu, Li-Juan Yi, Wei-Qing Chen, Xiu-E Li

**Affiliations:** ^1^Department of Nursing, Peking University School and Hospital of Stomatology, Beijing, China; ^2^Chongqing Key Laboratory of Translational Research for Cancer Metastasis and Individualized Treatment, Chongqing Cancer Institute, Chongqing Cancer Hospital, Chongqing University Cancer Hospital, Chongqing, China; ^3^Department of Nursing, Hunan Traditional Chinese Medical College, Zhuzhou, China

**Keywords:** head and neck cancer, radiotherapy, oral mucositis, zinc sulfate, systematic review

## Abstract

**Background:** Oral mucositis is an inevitable and distressing adverse event patients, who were treated with irradiation for head and neck cancer, face. Although several studies have investigated the potential of oral zinc sulfate in preventing radiation-induced oral mucositis in patients with head and neck cancers, conclusive results have not yet been found.

**Objective:** The aim of the present study is to determine whether oral zinc sulfate is effective in preventing radiation-induced oral mucositis, in patients with head and neck cancers.

**Methods:** We electronically searched all potential citations in PubMed, EMBASE, Cochrane Central Register of Controlled Trials (CENTRAL) and EBSCO from their inception to December 2018. After the search and checked literatures, extracted data and appraised risk of bias, RevMan software version 5.3 was used to perform meta-analysis.

**Results:** Four randomized controlled trials (RCTs) involving 162 patients were included. A meta-analysis showed that zinc sulfate did not decrease the incidence (RR [relative risk], 0.97; 95% CI [confidence interval], 0.74–1.28), and did not relieve the moderate and severe grade of radiation induced oral mucositis (moderate and severe oral mucositis: RR, 0.84; 95% CI, 0.14–4.87; severe oral mucositis: RR, 0.43; 95% CI, 0.00–38.98). A qualitative analysis suggested that zinc sulfate was associated with the onset of oral mucositis.

**Conclusions:** Based on limited evidence, zinc sulfate may not have the benefit of prophylaxis against radiation-induced oral mucositis, in patients with head and neck cancers. However, further RCTs with larger sample sizes and more rigorous methodologies are needed to enhance the evidence of these results.

## Introduction

Issued data revealed that head and neck cancer ranks eighth of all cancers, in incidence, and it has been estimated that about 710, 000 new cases were reported in 2018 (1). Radiotherapy, with or without chemotherapy, has always been considered as the medical regime that prevalently treats radical head and neck cancers (2, 3), however this regime may result in some distressing adverse side-effects (4). Of all of these adverse effects, oral mucositis (OM) seems to be the most common and troubling side effect, which results from the cytotoxic effects of radiation therapy on mucosa (5, 6). Approximately 60% of head and neck cancer patients who were instructed to receive radiotherapy will suffer from oral mucositis. More importantly, the incidence of oral mucositis increases to 90% when patients underwent concurrent chemotherapy (7). Head and neck cancer patients with oral mucositis, experience several distressing symptoms such as pain, as well as eating and swallowing difficulties (8) and more importantly, all symptoms related to oral mucositis will also worsen quality of life (QoL) (9, 10) and eventually alter the radiotherapy scheme (11, 12). Therefore, medical prophylaxis methods and treatment of radiation-induced oral mucositis have been explored, such as several organic products and low-level laser therapy (13). However, the efficacy of these regimes have not yet been completely established (14, 15).

Published evidence shows that zinc has the potential of relieving oxidant damage and the progression of reactive oxygen species (ROS)-induced disease (13, 16, 17). At present, published studies that evaluate the role of oral zinc sulfate in the prevention and treatment of radiation-induced oral mucositis in patients with head and neck cancers are limited (18–21). Among these studies, three showed that oral zinc sulfate reduced the severity of radiation-induced oral mucositis in patients with head and neck cancers (18, 20, 21); however Gorgu et al. did not find the potential of oral zinc sulfate in reducing the incidence and severity of radiation-induced oral mucositis (19). It therefore remains unclear whether oral zinc sulfate can prevent and treat radiation-induced oral mucositis. We consequently designed the present systematic review and meta-analysis to summarize all evidence to determine the role of oral zinc sulfate in the prevention and treatment of radiation-induced oral mucositis in patients with head and neck cancers.

## Methods

We designed this systematic review and meta-analysis in accordance with the requirements issued by the Cochrane Collaboration (CC) (22), and all results were reported according to the criteria listed in the Preferred Reporting Items for Systematic Reviews and Meta-Analysis (PRISMA) statement (23). We registered the present systematic review and meta-analysis in the International Prospective Register of Systematic Reviews (PROSPERO) platform and obtained an identifier CRD42018108533 (24). Moreover, we also published the full-text of our protocol in Medicine (13).

### Selection Criteria

The selection criteria was established as follows (13): (a) All adults, who were confirmed based on pathology, were administered radiotherapy with or without chemotherapy; (b) The treatment group received oral zinc sulfate and the control group took placebo capsules or received no treatment; (c) The incidence and severity of the oral mucositis were the primary outcomes, and the onset of oral mucositis and zinc-related adverse events were the secondary outcomes; and (d) Only randomized controlled trials (RCT) were considered as eligible for our study. Moreover, abstracts with sufficient data were also considered. The language of included studies was limited to English.

We excluded studies according to the following criteria: (a) patients previously underwent chemotherapy or radiotherapy, (b) patients had an infection of the mouth and a systemic infection, (c) previous oral mucositis, and (d) duplication with poor methodology and insufficient information.

### Definition

In the current study, incidence of radiation-induced oral mucositis was defined as the value of a number of oral mucositis cases, irrespective of grade, divided by the total number of cancer patients completing the whole study (25). The grade of oral mucositis was evaluated with a scoring system released by the Radiation Therapy Oncology Group (RTOG) or the Oral Mucositis Assessment Scale (OMAS) (26, 27). Grade 3 or 4 is considered severe and grade 2 moderate (28). The onset of oral mucositis was considered from the time of definitively being diagnosed with oral mucositis (13).

### Identification of Eligible Literature

We captured all relevant information by electronically searching PubMed, EMBASE, Cochrane Central Register of Controlled Trials (CENTRAL) and EBSCO from inception through to December 2018. We also manually checked the bibliographies of all eligible studies and topic-related reviews, in order to cover all potential citations. All search algorithms were established by combining Medical Subject Headings (MeSH) and text terms and updating these according to the requirements of each database (13). Two independent investigators were assigned to carry out all literature search strategies. The principal terms were “zinc,” “oral mucositis,” and “random.” The PubMed search algorithm is documented in Table 1. We used Endnote X7.0 software to manage all citations.

**Table 1:** Pubmed search algorithm.

**Search**	**Query**
#1	Search “Zinc Sulfate”[Mesh]
#2	Search (((((zinc sulfate[Text Word]) OR solvazinc[Text Word]) OR verazinc[Text Word]) OR zinc sulfate[Text Word]) OR zincomed[Text Word]) OR zincteral[Text Word]
**#3**	**Search #1 OR #2**
#4	Search “Mucositis”[Mesh] OR “Stomatitis”[Mesh]
#5	Search (((((((Mucositis[Text Word]) OR Mucositides[Text Word]) OR mucosa irritation[Text Word]) OR mucosa inflammation[Text Word]) OR Stomatitis[Text Word]) OR Stomatitides[Text Word]) OR Oromucositis[Text Word]) OR Oromucositides[Text Word]
**#6**	**Search #4 OR #5**
#7	Search “Randomized Controlled Trial” [Publication Type] OR “Randomized Controlled Trials as Topic”[Mesh]
#8	Search random*[Text Word]
**#9**	**Search #7 OR #8**
#10	Search #3 AND #6 AND #9

### Data Extraction

Two independent investigators were assigned to select the eligible studies based on the selection criteria (13). We then also assigned two investigators to independently extract data from all eligible studies (13). In the present study, the following information on lead author, publication year, age of participants, sample size, details of intervention regimes and outcomes of interest, were abstracted using the standard data extraction table designed by our team (13), which has been used in our previous systematic reviews and meta-analyses (29). A third author was available for consultation in order to solve differences in opinion of studies to consider for inclusion and the extraction of the basic information and data (13).

### Quality Assessment of Individual Study

Two independent reviewers were assigned to determine the bias risk from the following seven domains, including randomization sequence generation, allocation concealment, blinding of participants, blinding of study personnel, blinding of outcome assessors, incomplete outcome data, selective reporting, and other biases using the Cochrane risk of bias assessment tool (30, 31). The overall quality of the methodology of an individual study was graded according to the matched level between the actual information and the evaluation criteria (25). A third investigator was invited to solve any discrepancies between the two reviewers.

### Statistical Analysis

Continuous data were expressed as mean difference (MD) with 95% confidence intervals (CIs), and the dichotomous data were expressed as relative risk (RR) with 95% CIs. A random-effect model was selected to perform all statistical analyses within and between studies' heterogeneity simultaneously (32). The chi-square test and *I*^2^ statistic was used to qualitatively describe the heterogeneity and quantitatively estimate the proportion of the overall variation, respectively (33, 34). For a single outcome, we drew the funnel plot to qualitatively inspect the publication bias if the accumulated number of included studies was more than 10 (35). All analyses were performed using the RevMan 5.3 (Copenhagen, Denmark: The Nordic Cochrane Center, The Cochrane Collaboration, 2013).

## Results

### Identification and Selection of Eligible Studies

**Figure 1:**
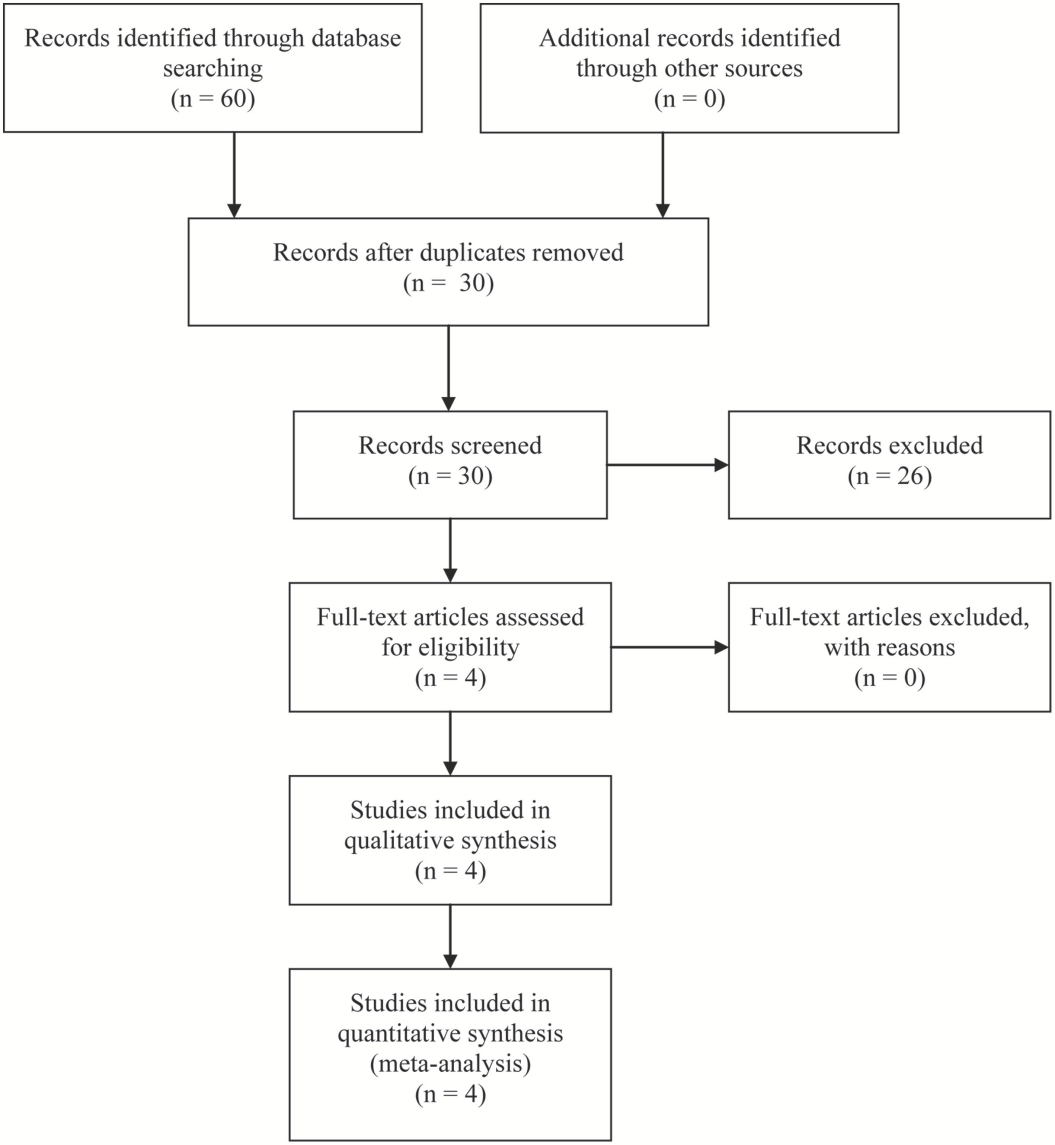
Flow chart of citation retrieval and selection.

A total of 60 studies were included at the initial stage of the literature search. After removing duplicate records based on the title and abstract of studies, 30 studies remained. Of 30 studies, nine related reviews and 17 studies on unrelated topics were excluded. After carefully review of the full text of the remaining four studies, THDR four studies were finally included in the qualitative and quantitative analysis (18–21). The flow diagram of citation retrieval and selection is shown in Figure 1.

### Summary of Included Study Characteristics

The basic characteristics of four studies are presented in Table 2. All studies were conducted in Iran or Turkey. In these four studies, 162 patients were recruited and the sample size in single trial ranged from 27 to 58. The median age of all participants was over 50 years old and the majority of patients were male. Oral administration was adopted in four studies, but the dose and frequency slightly differed. Moreover, only one study used the OMAS to evaluate the grade of oral mucositis (21). The other studies used the RTOG Acute Radiation Morbidity Scoring criteria (RTOG) to assess the grade of oral mucositis (18–20). All studies reported baseline information between zinc sulfate and the control groups had no statistically significant differences.

**Table 2:** The basic characteristics of the four included study.

**References**	**Country**	**Sample size (TG/CG)**	**Age (Median, years)**		**Gender (M/F)**		**Intervention**		**Scoring System**	**Outcomes**
			**TG**	**CG**	**TG**	**CG**	**TG**	**CG**		
Ertekin et al. (18)	Turkey	27 (15/12)	53 (36–69)	59 (18–71)	13/2	8/4	50 mg zinc sulfate capsule three times daily at 8-h intervals, from day 1 of RT until 6 weeks after RT	Placebo capsule three times daily at 8-h intervals	RTOG	Incidence of OM, OM situation 6 weeks after RT
Mosalaei et al. (20)	Iran	58 (29/29)	58.1	56.5	20/9	20/9	220 mg zinc sulfate capsule three times daily at 8-h intervals, from day 1 of RT until the end of RT	Placebo capsule three times daily at 8-h intervals	RTOG	Incidence of OM, Severity of OM
Gorgu et al. (19)	Turkey	40 (16/24)	56 (42–74)	58 (41–73)	15/1	24/0	4 zinc sulfate 25-mg tablets daily	No treatment	RTOG	Incidence of OM
Moslemi et al. (21)	Iran	37 (20/17)	49 (18–78)	52 (29–78)	12/8	9/8	30 mg zinc sulfate capsule three times daily at 8-h intervals, from 10 days before RT until 8 weeks after the end of RT	Placebo capsule three times daily at 8-h intervals	OMAS	Incidence of OM, Initiation and severity of OM

*TG, treatment group; CG, control group; RT, radiation therapy; M, Male; F, Female; RTOG, Radiation Therapy Oncology Group Acute Radiation Morbidity Scoring criteria; OMAS, Oral Mucositis Assessment Scale; OM, oral mucositis*.

### Quality Assessment of Eligible Studies

**Figure 2:**
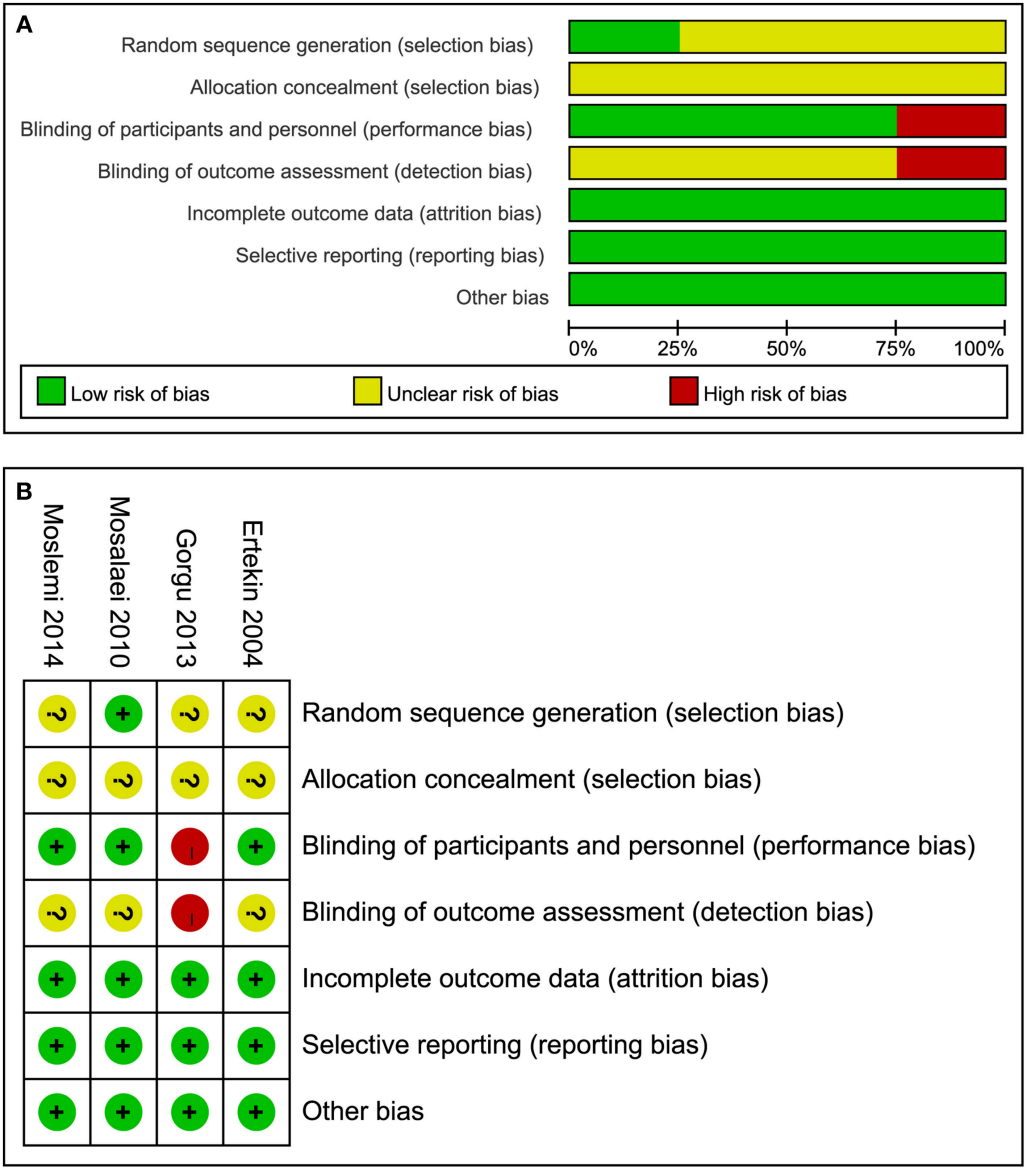
Risk of bias for all eligible studies: **(A)** risk of bias graph and **(B)** risk of bias summary.

All the studies included reported the use of the randomized method, but only Mosalaei et al. (20) described the randomized method in detail. Overall the studies included did not provide methods on how to conceal the allocation sequence. Of these four studies, one study did not take placebo capsules in the control group and did not report the blinding method used for participants not the personnel and outcome assessment, and was therefore evaluated as a high risk of bias (21). In summary, the overall quality of all eligible studies was considered as moderate. The detailed risk rating for individual studies and a summary of the risk ratings is presented in Figure 2.

### Incidence of Oral Mucositis

**Figure 3:**
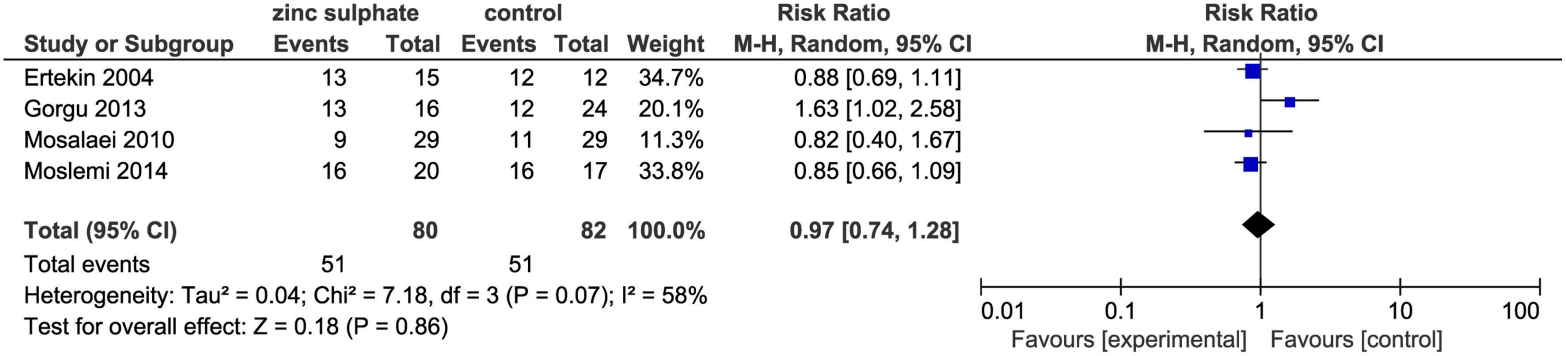
Meta-analysis of incidence of oral mucositis.

The four included trials (18–21) including 162 patients, reported the incidence of oral mucositis after the patients were instructed to take zinc sulfate daily. The meta-analysis suggested that there were no significant differences in the incidence of oral mucositis between the zinc sulfate and control groups (four RCTs; RR, 0.97; 95% CI, 0.74–1.28; *P* = 0.86; *I*^2^ = 58%; Figure 3).

### Severity of Oral Mucositis

**Figure 4:**
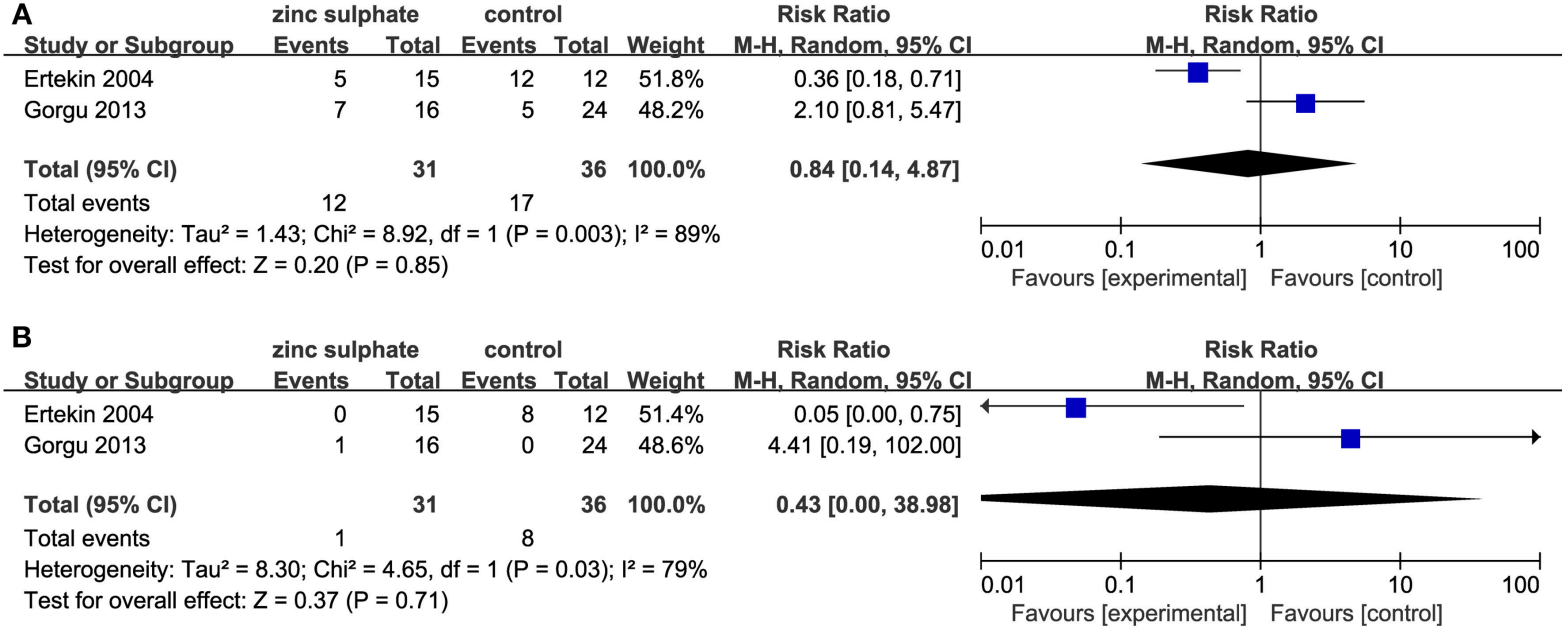
Meta-analysis of severity of oral mucositis: **(A)** Moderate and severe oral mucositis and **(B)** severe oral mucositis alone.

Among the four studies, two studies (18, 19) reported a moderate and severe grade of oral mucositis, reflecting the severity of oral mucositis. After performing a meta-analysis, the pooled results revealed that zinc sulfate had no impact on decreasing the severity of oral mucositis (Moderate and severe oral mucositis: RR, 0.84; 95% CI, 0.14–4.87; *P* = 0.85, *I*^2^ = 89%, Figure 4A; Severe oral mucositis: RR, 0.43; 95% CI, 0.00–38.98; *P* = 0.71, *I*^2^ = 79%, Figure 4B).

However, one study (20) reported the severity of oral mucositis by mean and standard deviation of the grade of oral mucositis, which could not be used in the meta-analysis. Therefore, a descriptive analysis was performed to clarify the findings. The study found that the mean severity score of oral mucositis was lower in the zinc group, which was only statistically significant in week 4, 5, and 6 (*P* = 0.02, 0.007, and 0.012 for weeks 4, 5, and 6). Another study (21) only showed a figure on the severity of mucositis in the placebo and zinc group over time. It suggested that in weeks 2 to 7 and 8, the severity of oral mucositis was lower in the zinc group (*p* < 0.003).

### Onset of Oral Mucositis

Of these four eligible studies, one (18) explored the onset of oral mucositis and showed that the onset of mucositis occurred in the 3rd week (0–5 weeks) and 2nd week (2–3 weeks) in the zinc sulfate group and the control group, respectively. The result indicated a significant difference between the two groups regarding the onset of mucositis (*p* < 0.05).

### Zinc Related Adverse Events

Of four studies, one study (18) reported an adverse zinc related event. The study reported RTOG Grade 3 vomiting and nausea developed in three patients in the zinc sulfate group.

### Publication Bias

In this systematic review, only the incidence and severity of oral mucositis could be included to perform a meta-analysis, but the accumulated number of eligible studies for these two outcomes were all <10. Therefore, we gave up on adopting a publication bias test based on a funnel plot and Egger test.

## Discussions

The rising prominence of head and neck cancers has become an important public health problem (13). Radiotherapy with or without chemotherapy is preferentially prescribed to treat head and neck cancers (13). However, because of the cytotoxic effect, radiotherapy inevitably brings about some adverse effects, of which oral mucositis is one of the most common and distressing (4). It has been noted that oral mucositis is associated with impaired oral functions, decreased quality of sleep, increases the risk of infections, and prolongs hospital stay (36). To date, several methods have been developed in order to prevent and treat radiation-induced oral mucositis, however, the efficacy and safety of these interventions have not been completely established. Thus, it is necessary to explore the potential of other alternatives to prevent and treat radiation-induced oral mucositis.

In the present systematic review and meta-analysis, we first elucidate the efficacy and safety of oral zinc sulfate to prevent and treat radiation-induced oral mucositis in patients with head and neck cancers. Our meta-analysis suggested that oral zinc sulfate could not significantly decrease the incidence of radiation-induced oral mucositis (RR, 0.97; 95% CI, 0.74–1.28). Additionally, the meta-analysis also found that oral zinc sulfate did not significantly alleviate the severity of oral mucositis (moderate and severe oral mucositis: RR, 0.84; 95% CI, 0.14–4.87; severe oral mucositis: RR, 0.43; 95% CI, 0.00–38.98). However, our qualitative analysis found that the onset of oral mucositis between the zinc sulfate and control groups was significantly different.

Ionizing radiation can directly damage cells, because of the chemical breaks in deoxyribonucleic acid (DNA). In addition, after interacting with water or oxygen molecules, radiation can produce reactive oxygen metabolites such as superoxide and hydrogen peroxide, which cause cell death through DNA damage (37). However, the oral mucosa has a very high cell turnover rate and so, constant epithelial replacement makes the oral mucosa vulnerable to the cytotoxic effects of radiation (38). Oral mucositis is simply the result of inflammatory changes in epithelial and subepithelial cells. Some experiments exactly demonstrate that zinc is not only an important element for multiple cellular activities, but also works as an organelle stabilizer and a stabilizer of the DNA structure, RNA and ribosome (13). Zinc can therefore accelerate wound healing. Zinc can also take part in the regulation of the immune system (18, 39).

Some researchers therefore hypothesized that zinc sulfate may be beneficial for oral mucositis in head and neck cancers and clinical trials were performed to test this hypothesis. However, our meta-analysis that found zinc sulfate did not decrease the incidence nor relieve the severity of radiation-induced oral mucositis. Of all the included studies in our meta-analysis, only the conclusion of Ertekin et al. (18) was consistent with our study, while the results of the other studies (18, 20, 21) were contradictory. It should be noted that in the study performed by Gorgu et al. (19), all eligible patients were assigned to receive four 25 mg zinc sulfate tablets daily, but patients in the other studies were assigned to take 30 mg zinc sulfate, 50 mg, or 220 mg capsules three times daily (18, 20, 21). Moreover, there was no treatment in the control group of the study performed by Gorgu et al. while the other studies assigned patients in the control group to receive a placebo which was similar in shape, taste, and color to the zinc sulfate capsules. These two aspects may therefore contribute to the inconsistency in results. After incorporating the four studies with different doses of zinc sulfate, we finally acquired the negative consequences. Although two published systematic reviews explored the potential of several interventions in preventing oral mucositis with cancers receiving treatment, and showed a trend in benefiting from zinc sulfate (40, 41), the two systematic reviews did not separately analyze the effect of zinc sulfate on radiation-induced oral mucositis. This limitation impaired the reliability of the conclusion that zinc sulfate had a positive effect on radiation-induced oral mucositis. We therefore think it is necessary to carry out a large-scale study with a similar zinc sulfate regime to explore the potential benefit of zinc sulfate in radiation-induced oral mucositis.

Because of the lack of specific data, the quantitative analysis could not be performed for the secondary outcomes and only a simple descriptive analysis was carried out. For the onset of oral mucositis, Ertekin et al. (18) offered positive evidence that there was a significant difference between the two groups regarding the onset of mucositis, and the time of occurrence of oral mucositis in the zinc sulfate group, was later than that of the control group. Published systematic reviews (40, 41) also found similar results showing that zinc sulfate may have the potential to prevent radiation-induced oral mucositis. Only Ertekin et al. (18) reported the occurrence of adverse events in the zinc sulfate group, but Ertekin et al. (18) did not perform a comparative analysis between the two groups. Moreover, adverse events were largely unremarkable in other published studies. Therefore, the safety of zinc sulfate needs to be considered cautiously.

Even though we included four studies with relatively large samples to perform a quantitative analysis, we have to acknowledge that some limitations still remain. On the one hand, we could not perform a subgroup analysis according to the dose of zinc sulfate because the number of eligible studies was insufficient. Thus, further studies should consider whether different doses of zinc sulfate influence the effect of zinc sulfate in radiation-induced oral mucositis. On the other hand, all the included studies were carried out in either Turkey or Iran. Therefore, the findings of our study should be cautiously interpreted when zinc sulfate is administered to patients with different genetic backgrounds.

## Conclusions

Based on the available evidence, it may be impossible for zinc sulfate to prevent and treat radiation-induced oral mucositis in patients with head and neck cancers. However, further RCTs with a larger cohort and more rigorous methodologies are required to improve the evidence of results, since the studies reviewed here caused limitations in the meta-analysis.

## Author Contributions

XT and TS conceived the study. L-JY and HC captured and selected citations. XT designed the data extraction table. W-QC and L-JY extracted data. TS and XT performed all statistical analyses and prepared the manuscript draft. XT, X-EL, and W-QC revised the initial manuscript. BS and X-LL supervised the revision of the manuscript. All authors approved the final version of manuscript.

### Conflict of Interest Statement

The authors declare that the research was conducted in the absence of any commercial or financial relationships that could be construed as a potential conflict of interest.
